# Acinar-to-Ductal Metaplasia Induced by Adenovirus-Mediated Pancreatic Expression of Isl1

**DOI:** 10.1371/journal.pone.0047536

**Published:** 2012-10-15

**Authors:** Satsuki Miyazaki, Fumi Tashiro, Junji Fujikura, Eiji Yamato, Jun-ichi Miyazaki

**Affiliations:** Division of Stem Cell Regulation Research, Osaka University Graduate School of Medicine, Suita, Osaka, Japan; University of British Columbia, Canada

## Abstract

Tubular complexes (TCs) are aggregates of duct-like monolayered cells in the developing and regenerating pancreas. Recent studies showed that TCs have regenerative potential, including islet neogenesis. We previously delivered adenovirus vector (AdV) into exocrine cells of the pancreas by intra-common bile ductal (ICBD) injection, and found that AdV expressing Pdx1, a pancreas-specific transcription factor, causes TC formation and islet neogenesis. We also established RTF-Pdx1-EGFP mice, which ubiquitously express Pdx1 when tetracycline is removed from the drinking water. However, exogenous Pdx1 expression in adult RTF-Pdx1-EGFP mice did not cause any pathological changes in the pancreas during three weeks of observation after tetracycline withdrawal. To examine whether the host immune response induced by AdV was involved in TC formation, we delivered AdVs expressing pancreas-related transcription factors or an irrelevant protein into the pancreas of RTF-Pdx1-EGFP mice. Histological analyses showed that both AdV injection and Pdx1 expression are required for TC formation. We also analyzed the effects of these ICBD-injected AdVs. AdV expressing Isl1, a proendocrine transcription factor, effectively induced TC formation through acinar-to-ductal metaplasia, and exogenous Pdx1 expression facilitated this process. Considering the regenerative potential of TCs, a strategy that efficiently induces TC formation may lead to novel therapies for diabetes.

## Introduction

Current therapies for type 1 diabetes, such as daily insulin injections, cannot prevent the progression of secondary complications. Only the transplantation of pancreas or islets can control blood glucose levels well enough to prevent these complications. However, given the shortage of available organs and the need for chronic immunosuppression, transplantation is not feasible as a general treatment. Attempts have been made to overcome these problems, including expanding patients’ existing β cells and differentiating embryonic stem (ES) cells into β cells, with only limited success. Another promising approach is the regeneration of β cells from other pancreatic cell types [1], [2].

After 90% pancreatectomy, adult rats show substantial pancreatic regeneration that is achieved by the replication of both pre-existing endocrine and exocrine cells and the proliferation of ducts, which subsequently differentiate into new pancreatic lobes [3], and increased islet neogenesis has also been reported in a number of other experimental systems [4], [5]. Interestingly, in all these studies, islet neogenesis is preceded by the formation of tubular complexes (TCs). TCs are duct-like tubes with a monolayer of cells lining the lumen [6], [7]. They have been observed during pancreatic regeneration after chemical or surgical injury in animal models and in human pancreatitis and pancreatic cancer [8]–[10]. Importantly, TCs have regenerative potential that includes islet neogenesis [11], [12]. Bonner-Weir *et al.* [1], [11] hypothesized that a rapidly replicating, mature duct cell transiently assumes a less-differentiated and less-restricted phenotype that can redifferentiate into any pancreatic cell type. Such plasticity would provide abundant multipotent progenitors in the adult pancreatic ducts for the normal renewal process [12]. Other studies suggest that adult pancreatic acinar tissue can transform into various pancreatic cell types through acinar-to-ductal transdifferentiation [13]–[15]. Regardless of this controversy, the ability to induce TC formation followed by duct-to-endocrine redifferentiation could lead to new therapies for diabetic hyperglycemia. To this end, it is important to clarify the molecular mechanisms underlying TC formation and subsequent islet neogenesis.

Pdx1 is a homeodomain-containing transcription factor that transactivates insulin gene expression through conserved enhancer elements; it is an essential regulator of pancreatic endocrine development and adult islet β-cell function [16]–[18]. In mice lacking Pdx1, pancreatic development is blocked at a very early stage [19]. In the developing pancreas, Pdx1 is first detected on embryonic day 8.5, in the foregut endoderm. By embryonic day 18.5, its expression is mostly restricted to mature β cells [20]. In animal models of pancreatic regeneration, Pdx1 is detected in the duct and duct-associated cells, suggesting that it is important for pancreatic regeneration and islet neogenesis [21], [22].

Adenovirus vectors (AdVs) are commonly used for *in vivo* gene transfer because of their high expression levels, high gene-transfer efficiency, and ease of high-titer production [23]. We previously reported effective AdV-mediated gene delivery into the pancreas by intra-common bile ductal (ICBD) injection [24], which transferred AdV mainly into exocrine cells close to major ducts. The ICBD transfer of Pdx1-expressing AdV (AdV-Pdx1) led to TC formation and islet neogenesis.

We recently established an RTF-Pdx1-EGFP mouse line [25], [26], in which Pdx1 and EGFP are expressed ubiquitously when tetracycline is removed from the drinking water. However, the expression of this exogenous Pdx1 in the pancreas of adult RTF-Pdx1-EGFP mice did not elicit histological changes during three weeks of observation after tetracycline withdrawal. This finding appeared inconsistent with our previous observation that transferring AdV-Pdx1 into the pancreas led to TC formation and islet neogenesis. Ferber *et al*. [27] reported that the AdV-mediated gene transfer of Pdx1 into mouse liver induced the expression of endogenous insulin 1 and 2. Interestingly, another group showed that insulin production in mouse liver required not only proendocrine transcription factor expression, but also inflammation in response to adenoviral packaging proteins [28]. Considering these reports, inflammation caused by AdV itself might contribute to TC formation and islet neogenesis in the pancreas.

In the present study, we delivered AdVs expressing pancreas-related transcription factors or an irrelevant protein by ICBD injection into the pancreas of RTF-Pdx1-EGFP mice induced to express Pdx1. Histological analysis showed that the delivery of any AdV led to TC formation and islet neogenesis in the pancreas. Among the AdVs examined, we found that Isl1-expressing AdV most effectively facilitated TC formation.

## Results

### Characteristics of the Pancreas of RTF-Pdx1-EGFP Mice

**Figure 1:**
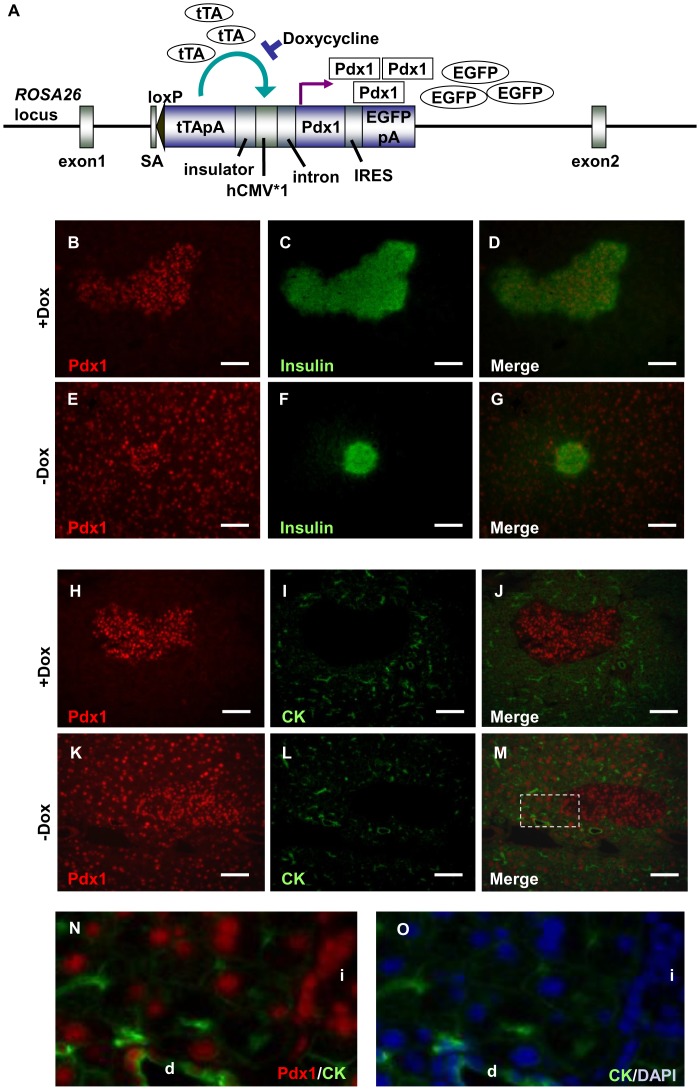
The inducible Pdx1 mouse model. (A) The construct of the Tet-off regulation unit for Pdx1 expression that was integrated into the *ROSA26* locus. The tetracycline transactivator gene is expressed under the control of the ROSA26 promoter. In knock-in mice (RTF-Pdx1-EGFP mice) heterozygous for the transgene, the continuous administration of Dox prevents the tTA from binding to the tetO, thereby inactivating the transcription of Pdx1 cDNA. After Dox is withdrawn, tTA binds to the tetO, transcribing the Pdx1 cDNA and EGFP cDNA. (B)–(M) Immunofluorescence analysis of Pdx1 (red) and insulin (green) (B–G) or Pdx1 (red) and cytokeratin 19 (CK) (green) (H–M) in pancreas sections from RTF-Pdx1-EGFP mice treated with Dox or untreated for 17 days. Bars = 100 µm. (N)–(O) Magnified view of the area surrounded by the dotted line in (M). Staining with anti-CK antibody (green) and DAPI (4, 6-diamidino-2-phenylindole) (blue) is shown in (O). “d” and “i” represent a duct and an islet, respectively.

RTF-Pdx1-EGFP mice ubiquitously express tetracycline-repressible transactivator (tTA) protein under the ROSA26 promoter, and this protein activates the downstream CMV*1 promoter to produce Pdx1 and EGFP, when Dox (doxycycline hydrochloride) is removed from the drinking water (**Figure 1A**) [25], [26]. This mouse line is maintained by continuously adding Dox to the water. As reported previously, all the main organs showed intense green fluorescence when Dox was withdrawn for more than 2 weeks [26]. We withdrew Dox from 7-week-old RTF-Pdx1-EGFP mice for 17 days, and then examined their pancreas (**Figure 1B–O**). As shown in **Figure 1G and 1M–O**, the nucleus of each pancreatic cell stained clearly with an anti-Pdx1 antibody, while only islet cells expressed endogenous Pdx1 in mice that were given Dox (**Figure 1D and 1J**). Since Pdx1 is thought to be essential for pancreatic development and regeneration, we examined hematoxylin and eosin-stained pancreatic sections for histological changes; however, none were seen three weeks after Dox withdrawal (**Figure S1**). This result appeared contradictory to our previous finding that the ICBD injection of Pdx1-expressing AdV causes extensive TC formation and insulin-positive cell regeneration in the pancreas [24]. However, AdV injection could have other effects, such as the induction of inflammatory responses, which might promote the regenerative changes in the pancreas. Therefore, we next examined the effects of ICBD injection of AdVs expressing pancreas-related transcription factors and a control AdV expressing an irrelevant protein into RTF-Pdx1-EGFP mice.

### AdV-mediated Gene Delivery by ICBD Injection

**Figure 2:**
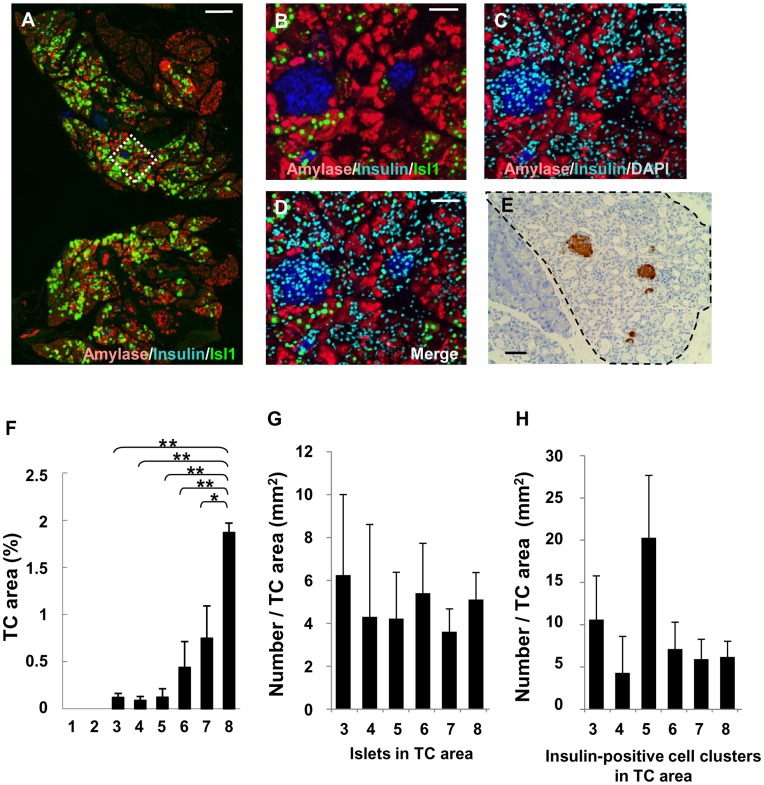
Quantification of histological changes in the AdV-infected mouse pancreas. (A)–(D) Sections of pancreas excised 1.5 days after injection were stained with an anti-Isl1 antibody (green), anti-amylase antibody (red), and anti-insulin antibody (blue). Bar = 300 µm. Magnified view of the area surrounded by the dotted line in (A) is shown in (B)–(D). Staining with DAPI (blue) is included in (C) and (D). Bar = 50 µm. (E) Pancreatic section stained with an anti-insulin antibody (brown) and counterstained with hematoxylin. The area surrounded by the dotted black line shows TCs. Bar = 100 µm. (F)–(H) Quantification of histological changes. Mice were divided into eight groups: Group 1, RTF-Pdx1-EGFP mice mock-injected after Dox withdrawal (n = 5); group 2, wild-type mice given AdV-CAT (n = 5); group 3, RTF-Pdx1-EGFP mice given AdV-CAT after Dox withdrawal (n = 4); group 4, RTF-Pdx1-EGFP mice given AdV-Ptf1a after Dox withdrawal (n = 3); group 5, RTF-Pdx1-EGFP mice given AdV-Neurod1 after Dox withdrawal (n = 3); group 6, wild-type mice given AdV-Isl1 (n = 5); group 7, RTF-Pdx1-EGFP mice given AdV-Isl1 in the presence of Dox (n = 5); group 8, RTF-Pdx1-EGFP mice given AdV-Isl1 after Dox withdrawal (n = 6). Six days after AdV-Isl1 injection, the pancreata were removed for immunohistochemical analysis. Three nonconsecutive sections from each pancreas were stained with an anti-insulin antibody followed by hematoxylin counterstaining, and examined by light microscopy. Each section was digitally scanned and the image files were analyzed by NIH image to calculate the percentage of the area that was occupied by TCs, as described in **Materials and Methods**. The islets and insulin-positive cell clusters consisting of 1–4 cells in the TC area on each section were counted. Percentage of TC area/total section area is shown in (F). The numbers of islets and insulin-positive cell clusters/TC area (mm^2^) are shown in (G) and (H), respectively. Values are means ± SE (*P<0.05 and **P<0.01 by Student’s *t*-test).

AdVs expressing Isl1, Ptf1a, Neurod1, or CAT (chloramphenicol acetyltransferase) as an irrelevant protein were prepared. Dox was withdrawn from 9- to 10-week-old RTF-Pdx1-EGFP mice for 14 to 17 days, and each AdV was administered by ICBD injection, as described previously [24]. **Figure 2A**–**D** shows immunostained sections of a pancreas excised 1.5 d after AdV-Isl1 injection. Many Isl1-positive cells were observed in the exocrine pancreas, indicating efficient transduction. We examined the histological changes of the pancreata from eight groups (n = 3 to 6): Group 1, RTF-Pdx1-EGFP mice mock-injected after Dox withdrawal; Group 2, wild-type mice given AdV-CAT, Group 3: RTF-Pdx1-EGFP mice given AdV-CAT after Dox withdrawal, Group 4: RTF-Pdx1-EGFP mice given AdV-Ptf1a after Dox withdrawal, Group 5: RTF-Pdx1-EGFP mice given AdV-Neurod1 after Dox withdrawal, Group 6: wild-type mice given AdV-Isl1, Group 7: RTF-Pdx1-EGFP mice given AdV-Isl1 during Dox administration, and Group 8: RTF-Pdx1-EGFP mice given AdV-Isl1 after Dox withdrawal. The pancreata from mice given AdVs were excised 6 days after the injection and cut into sections. Three sections from each mouse were stained with an anti-insulin antibody, counterstained with hematoxylin, and examined.

The area surrounded by the dotted black line in **Figure 2E** shows the TCs. The percentage of each section’s area taken up by the TCs (TC percent) was calculated (see **Materials and Methods**) **(** **Figure 2F** **)**. No TCs were detected in the pancreas of wild-type mice injected with AdV-CAT (Group 2) or of RTF-Pdx1-EGFP mice mock-injected after Dox withdrawal (Group 1). In contrast, the pancreata from RTF-Pdx1-EGFP mice given any of the AdVs after Dox withdrawal showed TC formation (Groups 3, 4, 5, and 8). TCs were found preferentially in the proximal part of the pancreas, where efficient transduction by the AdVs occurred. These results supported the idea that a combination of Pdx1 expression and AdV-mediated inflammatory stimuli could lead to regenerative changes in the pancreas. The TC percent in the pancreas of RTF-Pdx1-EGFP mice injected with AdV-Isl1 after Dox withdrawal (Group 8) was much greater than in those injected with AdV-CAT (Group 3), AdV-Ptf1a (Group 4), or AdV-Neurod1 (Group 5) (1.87±0.10% vs 0.12±0.04%, 0.09±0.04%, or 0.12±0.04%, respectively; *P*<0.01). The TC percent in the pancreas of wild-type mice and that of RTF-Pdx1-EGFP mice under Dox treatment were both increased following treatment with AdV-Isl1 (0.44±0.27% (Group 6) and 0.75±0.34% (Group 7), respectively). Thus, among the AdVs examined, AdV-Isl1 caused the most extensive occurrence of TCs. The observation that even wild-type mice showed TC formation with AdV-Isl1, but not with AdV-CAT, indicated that TCs could be induced specifically by AdV-Isl1 in the absence of Pdx1.

We next counted the numbers of islets and insulin-positive cell clusters composed of 1–4 cells in the TC area. As shown in **Figure 2G and 2H**, the numbers of islets and insulin-positive cell clusters relative to the area occupied by TCs were not significantly different among Groups 3–8. Thus, AdV-mediated Isl1 delivery into the Pdx1-expressing pancreas effectively induced TC formation, which was accompanied by islet neogenesis in the TC area.

### Analysis of the Cells in the TC Area

To examine the molecular mechanisms underlying the induction of TC formation by Isl1, we performed immunofluorescence analyses of the pancreas 1.5, 3, and 6 days after ICBD injection of AdV-Isl-1. In normal adult mice, Isl1 expression is weak and restricted to the pancreatic islet cells [29]. In contrast, in mice that received AdV-Isl1, the pancreas included cells that were strongly positive for Isl1, which were mostly acinar cells (**Figure 2A–D**). It should be noted that, in **Figure 2A–D**, the islet cells appeared negative for Isl1, but a longer exposure showed that they were positive for it (**Figure S2**).

**Figure 3:**
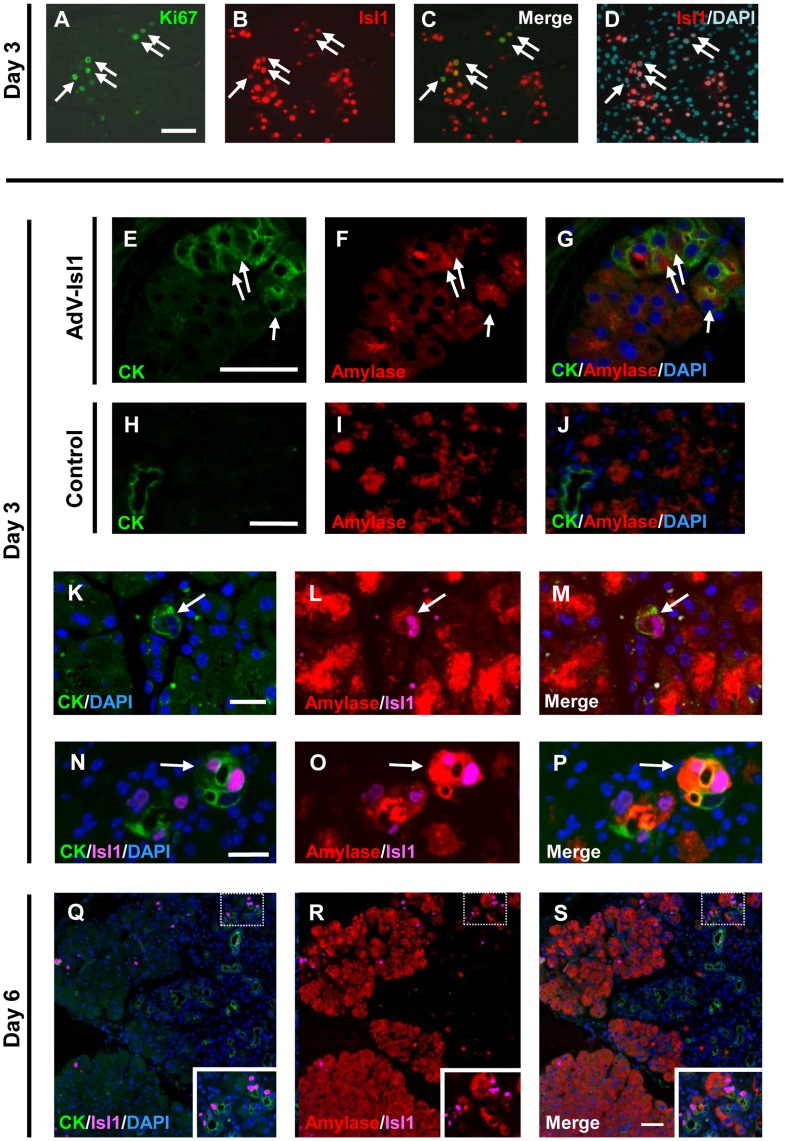
Immunofluorescence analysis of transdifferentiation in the AdV-Isl1-infected RTF-Pdx1-EGFP mouse pancreas. (A)–(D) Pancreas was excised 3 days after injection. Pancreatic sections were stained with anti-Ki67 (A, green) and anti-Isl1 (B, red). Merged view of (A) and (B) is shown in (C). Staining with anti-Isl1 (red) and DAPI (light blue) is shown in (D). Arrows indicate doubly stained cells. Note that most of the Ki67-positive cells were Isl1-positive. Bar = 100 µm. (E)–(G) Sections from AdV-Isl1-infected mouse pancreas, excised 3 days after injection, were stained with anti-CK (E, green) and anti-amylase (F, red). Merged view of (E) and (F) is shown in (G) with DAPI (blue). Arrows indicate CK and amylase double-positive cells. Bars = 50 µm. (H)–(J) Control pancreatic sections from wild-type mice not given an AdV injection were stained with anti-CK (H, green) and anti-amylase (I, red). Merged view of (H) and (I) is shown in (J) with DAPI (blue). Note that the duct was clearly stained with anti-CK but not with anti-amylase. Bars = 50 µm. (K)–(P) Pancreatic sections from AdV-Isl1-infected mice were stained with anti-CK (green), anti-Isl1 (magenta), and anti-amylase (red) with DAPI (blue). Merged image of (K) and (L) is shown in (M). Merged image of (N) and (O) is shown in (P). Arrows indicate CK/Isl1/amylase triple positive cells. Bar = 50 µm. (Q)–(S) Immunofluorescence analysis of the pancreas from mice given AdV-Isl1. Pancreas was excised 6 days after injection. Pancreatic sections were stained with anti-CK (green) and anti-Isl1 (magenta) with DAPI (blue) (Q) or with anti-amylase (red) and anti-Isl1 (magenta) (R). Merged view of (Q) and (R) is shown in (S). Note that most of the Isl1-positive cells in TCs were CK-positive. Bar = 50 µm.

Previous studies showed that cell turnover in the TC region is much more active than in the non-TC region, mainly owing to increased cell proliferation and the apoptosis of duct-like cells [12]. To determine whether the cells infected with AdV-Isl1 showed increased proliferation, we examined the proliferation marker Ki67 in the pancreas of RTF-Pdx1-EGFP mice 3 days after the ICBD injection of AdV-Isl1 after Dox withdrawal, when TCs had not yet formed in the pancreas. We frequently found Ki67 positive cells among Isl1 positive cells in the exocrine pancreas (495/2739 Isl1 positive cells; 18.1%) (**Figure 3A–D**), suggesting that the AdV-mediated delivery of Isl1 induced the proliferation of acinar-derived cells, leading to TC formation.

We next examined whether this TC induction by Isl1 occurred through the transdifferentiation of acinar cells into duct cells or by ductal-cell replacement owing to acinar cell loss. If the TCs were formed by acinar-to-ductal transdifferentiation, intermediate cells expressing both acinar and duct-cell markers should be present. We performed an immunofluorescence analysis 3 days after the injection, and observed cells that were double labeled for amylase, a marker for exocrine cells, and cytokeratin (CK), a marker for ductal epithelium, in the pancreas of mice given AdV-Isl1 (**Figure 3E–G**). In the normal control pancreas, CK was located only in duct cells, and amylase only in exocrine cells; no double-labeled cells were observed (**Figure 3H–J**). These results suggested that the TC formation was attributable to acinar-to-ductal transdifferentiation.

To examine the relationship between exogenous Isl1 expression and the acinar-to-ductal transdifferentiation, sections were stained with antibodies to Isl1, CK, and amylase. The results showed the occurrence of CK/amylase/Isl1 triple-positive cells (**Figure 3K–M and 3N–P**), although these cells were found only infrequently. To analyze this process, we performed immunofluorescence analysis 6 days after the injection. At this time point, the number of Isl1-positive cells had decreased due to transient nature of AdV-mediated gene expression and Isl1-positive cells in the TC area were mostly CK-positive (**Figure 3Q–S**), but negative for amylase. These results suggested that the AdV-mediated Isl1 expression directly induced acinar-to-ductal transdifferentiation and that cells expressing both CK and amylase existed only transiently.

**Figure 4:**
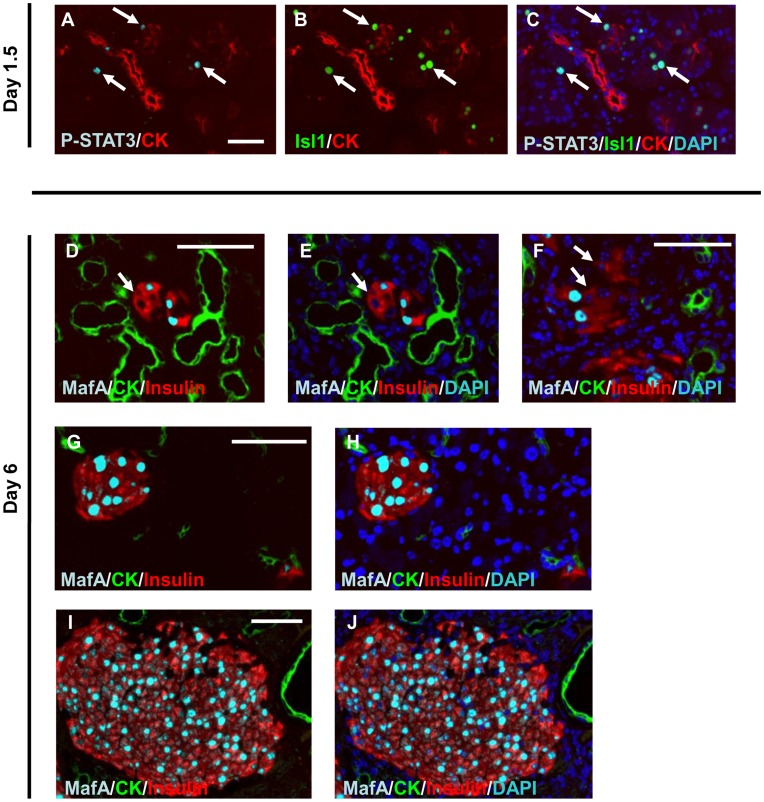
Immunofluorescence analysis of the pancreas of RTF-Pdx1-EGFP mice given AdV-Isl1. (A)–(C) Pancreas was excised 1.5 days after AdV injection, and sections were stained with anti-CK (red), anti-phospho-STAT3 (light blue), and anti-Isl1 (green). Merged view of (A) and (B) is shown in (C) with DAPI (blue). Arrows indicate cells stained with both anti-Isl1 and anti-phospho-STAT3 antibodies. Bar = 100 µm. (D)–(F) Expression of MafA in the TC area. Sections from AdV-Isl1-injected RTF-Pdx1-EGFP mouse pancreas, excised 6 days after the injection, were stained with anti-insulin (red), anti-MafA (light blue), and anti-CK (green). Merged view of (D) and DAPI staining (blue) is shown in (E). Another part of the TC area is shown in (F) with DAPI staining. Heterogeneous staining for MafA was seen in the insulin-positive cells in the TC area. Arrows indicate insulin-positive, but MafA-negative cells. (G)–(J) Expression of MafA in the non-TC area of the same pancreatic section as (D–F). Merged view of (G) and DAPI staining is shown in (H). Merged view of (I) and DAPI staining is shown in (J). Almost all the insulin-positive islet cells were positive for MafA. Bars = 50 µm.

Recently, Isl1 was shown to elevate the tyrosine phosphorylation, DNA-binding activity, and target gene expression of signal transducer and activator of transcription (STAT)3 [30]. STAT3 is activated in cells within the ductal structures in human pancreatic cancer and in a mouse model of this cancer [31], [32]. Thus, it is possible that the ductal proliferation we observed was caused by Isl1’s promotion of STAT3 activation in the pancreatic cells. We therefore examined the expression of phosphorylated STAT3 in the pancreas 1.5 days after the AdV-Isl1 injection. Phosphorylated STAT3 was detected in the nucleus of acinar cells, which were also positive for Isl1 (**Figure 4A–C**). Phosphorylated STAT3 was not detected without AdV-Isl1 injection (not shown). Our results suggested that Isl1 induced STAT3 phosphorylation, leading to the proliferation of acinar-derived ductal cells and TC formation.

### Analysis of the Islets and Insulin-positive Cell Clusters in the TC Area

It was recently reported that MafA, a proendocrine transcription factor, regulates insulin gene expression in β cells [33]–[37]. In adults, MafA’s expression is highly restricted to β cells. Therefore, we examined the expression of MafA by immunohistochemical analysis of the pancreas of the RTF-Pdx1-EGFP mice given AdV-Isl1 after the withdrawal of Dox. Interestingly, insulin-positive cells in the TC region were often negative for MafA (**Figure 4D–F**), whereas those in the non-TC region were almost completely positive (**Figure 4G–J**). These observations suggested that the insulin-positive cells in the TC region might have different characteristics from the insulin-positive cells in the non-TC area, supporting the idea that the insulin-positive cells in the TC region are newly induced and immature.

## Discussion

The transgenic expression of Pdx1 in the pancreas of adult mice did not cause any pathological changes in this organ during three weeks of observation, which appeared inconsistent with our previous observation that the transfer of AdV-Pdx1 into the pancreas led to TC formation and islet neogenesis. However, the ICBD injection of AdV expressing an irrelevant protein into the pancreas expressing transgenic Pdx1 also induced TC formation and islet neogenesis. These results suggest that inflammation caused by the AdV particles plays an indispensable role in TC formation and islet neogenesis in the pancreas. Wang *et al.* [28] showed that a host response to adenovirus combined with the expression of a pro-endocrine pancreas transcription factor is required to induce insulin production in the liver of diabetic mice. Thus, inflammation induced by AdV may stimulate a specific signaling pathway that leads to regenerative responses.

In the current study, we also delivered AdVs expressing pancreas-related transcription factors into the pancreas of RTF-Pdx1-EGFP mice with the hope of enhancing regeneration. Of the factors tested, we found that Isl1-expressing AdV caused the most prominent effects. Isl1 belongs to the LIM homeodomain transcription factor family. It has roles in the differentiation of motor neurons and organogenesis of the pancreas and heart, although little is known about its regulatory mechanism and target genes. In embryos, Isl1 is expressed in the dorsal pancreatic mesenchymal cells, but not in the ventral pancreatic epithelium. Isl1-deficient embryos show a complete loss of differentiated islet cells [38]. Recently, Hao *et al.* [30] identified interactions between Isl1 and Janus tyrosine kinase (Jak), as well as STAT3 in mammalian cells. They found that Isl1 not only forms a complex with Jak1 and STAT3, but also triggers the tyrosine phosphorylation of Jak1 and its kinase activity, thereby elevating the tyrosine phosphorylation, DNA-binding activity, and target gene expression of STAT3. In light of these reports, our results suggest that Isl1 expression may induce STAT3 activation in pancreatic cells, leading to ductal proliferation. These proliferating cells seem to share characteristics with a subpopulation of pancreatic endocrine precursor cells that express CK19 but no endocrine hormones during pancreatic development [39]. We previously reported the isolation and characterization of mouse Pdx1-positive multipotent duct-derived cells [40]. We recently analyzed the transcription factor cascade that induces endocrine and exocrine cell lineages from these cells, which showed that exogenous Isl1 expression effectively promoted their differentiation into endocrine cell lineages [41]. These reports and our current study suggest novel critical roles for Isl1 in the differentiation and proliferation of pancreatic precursor cells.

Miyatsuka *et al.* [42] reported a transgenic mouse model in which Pdx1 is constitutively expressed in all the pancreatic lineages. They showed that persistent Pdx1 expression cell-autonomously induces an acinar-to-ductal transition that is dependent on STAT3 activation. This appears to contrast with our observation that Pdx1 expression alone did not cause any obvious pathological changes in the pancreas, but this discrepancy is probably accounted for by the differences in the developmental stage and in the duration of Pdx1 expression. In fact, when Dox was withheld from the drinking water from the time of conception, the ERTF-Pdx1-EGFP progeny mice showed severe dysmorphogenesis of the exocrine pancreas at the age of 2 weeks (not shown), which was very similar to that observed in the above report [42].

Another important observation from this study is that β cells negative for MafA were often seen exclusively in the TC region after AdV-Isl1 injection. MafA is a member of the L-Maf family of basic leucine zipper transcription factors, and it binds to RIPE3b, an enhancer of the insulin 1 and 2 genes. Recent studies indicate that MafA is a β-cell-specific and glucose-regulated transcriptional activator for insulin gene expression [33]–[36]. Nishimura *et al.* [43] analyzed the expression pattern of L-Maf factors in the pancreas of embryonic and adult mice to examine their role in the differentiation of pancreatic endocrine cells. Their results suggest that the differentiation of β cells proceeds through a MafB(+) MafA(−) Insulin(+) intermediate cell before becoming a MafB(−) MafA(+) Insulin(+) cell. The present report raises the possibility that MafA-negative islet cells in the regenerating TCs represent newly induced, functionally immature β cells that resemble the β cells seen during development, although there may be multiple causes for the reduction in MafA expression [44].

Zhou *et al.* [45] reported that the AdV-mediated introduction of three transcription factors, Ngn3, Pdx1, and MafA, into the differentiated pancreatic exocrine cells of adult mice, reprograms them to become β-like cells. Considering that ductal proliferation was not seen after the AdV infection in that study, their findings may show the direct cellular reprogramming of exocrine cells into β cells by a mechanism very different from the one underlying our current results. In their experiments, the first β cells appeared 3 days after the AdV infection, as isolated cells or in small clusters. By contrast, we detected insulin-positive cell clusters mainly in the TC area, and these clusters may originate from the TCs. This is consistent with the idea that islet endocrine cells are predominantly derived from the precursor cells that reside among pancreatic epithelial duct cells or duct-associated cells both during embryonic development and later in life [46].

In conclusion, we demonstrated that AdV injection combined with the expression of a pro-endocrine transcription factor is required to induce TC formation and islet neogenesis, and that the AdV-mediated gene transfer of Isl1 into the Pdx1-expressing pancreas effectively induced these pathological changes. Our results suggested that the TC formation was attributable to acinar-to-ductal transdifferentiation, but lineage tracing studies will be required to determine the origin of the cells in the TC region. Although we could not demonstrate directly that the insulin-positive cells in the TC area were newly regenerated, the AdV-mediated transfer of Isl1 into the Pdx1-expressing pancreas may be a useful strategy for studying pancreatic endocrine neogenesis, and could contribute to a gene therapy to cure diabetes.

## Materials and Methods

### Ethics Statement

All experiments involving animals were carried out in accordance with institutional guidelines under the protocols (No. 414 and No. 19-055-0), which were approved by the Animal Care and Use Committee of the Osaka University Graduate School of Medicine.

### Pdx1-expressing Mice

The RTFN-Pdx1-EGFP mouse line was described previously [25], [26]. This line was maintained by crosses with C57BL/6J mice. RTFN-Pdx1-EGFP males were crossed with CAG-Cre transgenic females to remove the *loxP*-flanked neor gene [47]. The resulting progeny, designated RTF-Pdx1-EGFP, had lost the neo^r^ gene from the *ROSA26* locus [48], and their Tet-off system was active. These mice were maintained under the continuous administration of Dox (doxycycline hydrochloride; Sigma-Aldrich, St. Louis, MO) in the drinking water at 0.05 mg/ml in light-protected bottles, to suppress the Pdx1 expression. Seven to ten week-old RTF-Pdx1-EGFP mice and their transgene-negative littermates were used in the present study.

### Construction of AdVs

The cDNAs for Isl1, Ptf1a, and Neurod1/BETA2 were cloned from mouse tissues or the mouse insulinoma cell line MIN6 [49]. AdVs expressing these cDNAs were generated according our previously reported method [50] and designated AdV-Isl1, AdV-Ptf1a, and AdV-Neurod1, respectively. We also prepared a control vector, AdV-CAT, expressing chloramphenicol acetyltransferase (CAT). The AdVs were propagated in 293 cells. Infected cells were collected by centrifugation and subjected to six cycles of freezing and thawing. The resulting cell lysates were spun, and the supernatants were titrated using the AdenoX Rapid Titer Kit (BD Biosciences, San Jose, CA) and used for the following experiments.

### ICBD Injection of AdVs

Mice were subjected to laparotomy under general anesthesia with pentobarbital. They then received an injection of 300 µl of AdV solution (5–10×10^8^ IFU) into the common bile duct through a 27- or 29-G needle [24]. The mice were sacrificed 1.5–7 days after the injection.

### Histochemical Analysis

Pancreatic tissue was fixed in 20% formaldehyde and processed for paraffin embedding. For anti-insulin immunohistochemical staining, sections of paraffin-embedded pancreatic tissue (5-µm thick) were deparaffinized, dehydrated, and incubated with 10% normal goat serum in phosphate buffered saline (PBS) and then with a diluted anti-human insulin antibody that is also reactive with mouse insulin (Oriental Yeast Co., Tokyo, Japan). The sections were next incubated with biotinylated goat anti-rabbit immunoglobulin (Dako, Carpinteria, CA) and then with peroxidase-conjugated streptavidin (Dako). After incubation in a DAB solution (20 mg of 3, 3′-diaminobenzidine tetrahydrochloride and 5 µl of 30% hydrogen peroxide in 100 ml PBS), the sections were counterstained with hematoxylin, dehydrated, and examined.

### Immunofluorescence Analysis

Paraffin sections of pancreatic tissue were processed as described above. For frozen sections, isolated pancreas was embedded in OTC compound (Tissue-TEC, Miles, Elkhart, IN), frozen in liquid nitrogen, and then cut into 7-µm-thick frozen sections on a cryostat. The frozen sections were placed on slides, and fixed in 4% paraformaldehyde or 15% formaldehyde for 10 min. After fixation, the cells were rinsed with PBS, incubated for 5 min in cold methanol, and after a second wash, incubated in blocking reagent. For both paraffin sections and frozen sections, the samples were incubated with the first antibody at 4°C overnight and then with a fluorescein-conjugated second antibody for 60 min at room temperature. After each antibody incubation, the sections were washed in PBS for 5 min with three changes. The first antibodies were guinea pig anti-insulin antibody (Dako), mouse anti-pancytokeratin antibody (Sigma-Aldrich), mouse anti-Ki67 antibody (BD Pharmingen, San Diego, CA), rat anti-cytokeratin 19 monoclonal antibody (TROMA III), mouse anti-Isl1 monoclonal antibody (40.2D6 and 39.4D5) (Developmental Studies Hybridoma Bank, Iowa City, IA), rabbit monoclonal antibody against STAT3 phosphorylated at Tyr705 (Cell Signaling, Beverly, MA), rabbit anti-amylase antibody (Sigma-Aldrich), rabbit anti-Pdx1 antibody (Transgenic, Kumamoto, Japan), and rabbit anti-MafA antibody (Bethyl Laboratories Inc., Montgomery, TX). The second antibodies were Alexa Fluor 488- and 568-conjugated anti-mouse IgG1, Alexa Fluor 568-conjugated anti-mouse IgG2b, Alexa Fluor 568-conjugated anti-rabbit IgG, Alexa Fluor 488-, 594-, and 647-conjugated anti-guinea pig IgG, and Alexa Fluor 568-conjugated anti-rat IgG (Molecular Probes, Eugene, OR). To detect phosphorylated STAT3 and MafA, we used the TSA system (Perkin Elmer, Norwalk, CT) in combination with HRP (horseradish peroxidase)-conjugated goat anti-rabbit IgG and streptavidin-Alexa 647 for signal amplification, according to the manufacturer’s instructions. The sections were observed by immunofluorescence microscopy (Keyence, Osaka, Japan; Olympus, Tokyo, Japan).

### Evaluation of Histological Changes

Each AdV was administered to three to six mice by ICBD injection. The whole pancreas was excised, coiled up (so that both the proximal and distal parts of the pancreas could be examined on the same section), and placed on a piece of waxed paper. Three nonconsecutive paraffin sections, which were as large as possible for each pancreas, were immunohistochemically stained with an anti-insulin antibody and examined by light microscopy. Each section was digitally scanned, and the image files were analyzed by NIH Image to calculate the percentage of the area that was occupied by TCs. The numbers of islets and of insulin-positive cell clusters in the TC area were also counted and divided by the area occupied by TCs. Insulin-positive cell clusters were defined as those consisting of 1–4 cells. The statistical analysis was performed by Student’s *t-*test. The *P*-values for significance were set to 0.05. Data are expressed as means ± SE.

## Supporting Information

Figure S1 **Histological analysis of the RTF-Pdx1-EGFP mouse pancreas.** Pancreas was excised from the RTF-Pdx1-EGFP mouse 3 weeks after Dox withdrawal, and a pancreas section was stained with hematoxylin-eosin. Bar = 100 µm.
(PDF)

Figure S2 **Detection of Isl1 in the islets of the mouse pancreas.** Pancreas section of a wild-type mouse was stained with an anti-Isl1 antibody (green), anti-amylase antibody (magenta), and anti-insulin antibody (red). Bars = 50 µm.
(PDF)
