# Supplementary figures and images for: Acinar-to-Ductal Metaplasia Induced by Adenovirus-Mediated Pancreatic Expression of Isl1
Source: PLoS One. 2012 Oct 15;7(10):e47536. doi: 10.1371/journal.pone.0047536 (PMC3471997; doi:10.1371/journal.pone.0047536)

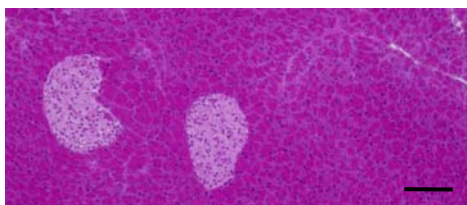

Supplement: Figure S1 — Histological analysis of the RTF-Pdx1-EGFP mouse pancreas. Pancreas was excised from the RTF-Pdx1-EGFP mouse 3 weeks after Dox withdrawal, and a pancreas section was stained with hematoxylin-eosin. Bar = 100 µm. (PDF) [file pone.0047536.s001.pdf]

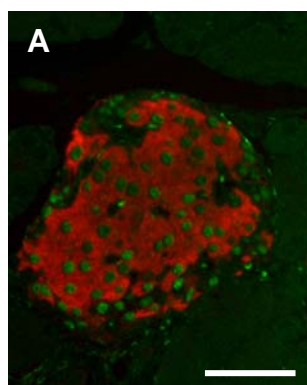

**Isl1/Insulin**

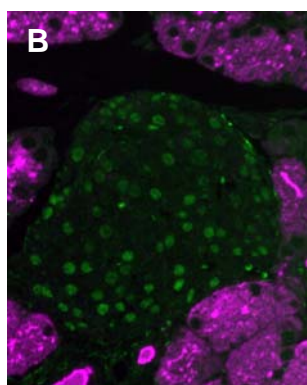

**Isl1/Amylase**

Supplement: Figure S2 — Detection of Isl1 in the islets of the mouse pancreas. Pancreas section of a wild-type mouse was stained with an anti-Isl1 antibody (green), anti-amylase antibody (magenta), and anti-insulin antibody (red). Bars = 50 µm. (PDF) [file pone.0047536.s002.pdf]
